# Assessment of Maternal Serum Biochemical Attributes and Fetal Ultrasound Scans in First-Trimester Low-Risk Noninvasive Prenatal-Tested Pregnant Women

**DOI:** 10.7759/cureus.24653

**Published:** 2022-05-01

**Authors:** J R Keshari, Uday Kumar, Prabhat Kumar, Dipali Prasad, Pritam Prakash, Pushpanjali P

**Affiliations:** 1 Biochemistry, Indira Gandhi Institute of Medical Sciences, Patna, IND; 2 Pathology, Indira Gandhi Institute of Medical Sciences, Patna, IND; 3 Obstetrics and Gynaecology, Indira Gandhi Institute of Medical Sciences, Patna, IND

**Keywords:** crl, first trimester, nuchal translucency, nipt, afp, papp-a

## Abstract

Introduction: The first trimester of pregnancy is marked by several important disrupting changes as a result of complex biological upshot of events required for the development of the fetus. These changes in the biological events result in changes in the maternal serum biomarkers that are associated with fetal growth. The aim of the present study was to evaluate the correlation between the maternal blood biochemical determinants such as pregnancy-associated plasma protein-A (PAPP-A) and alpha fetoprotein (AFP) with ultrasound scans during early pregnancy in the first trimester.

Methods: The study included 139 women whose fetus was alive between 11±1 weeks of gestation. The risk of fetal chromosomal abnormalities at first trimester was analyzed by the VeriSeq NIPT Solution v2 platform (Illumina, San Diego, CA, USA) for noninvasive prenatal testing (NIPT) assay. The PAPP-A and AFP levels were evaluated by chemiluminescent immunoassays. The levels of PAPP-A and AFP were correlated with the fetal heart rate (HR), crown rump length (CRL), and nuchal translucency (NT) by Pearson’s correlation analysis.

Results: The mean age of the participants was 28.35±3.87 years (minimum=21, maximum=35). The mean AFP and PAPP-A levels in the maternal plasma were 14.76±1.04 ng/mL and 4.37±0.86 mIU/ml respectively. The mean FHR, CRL, and NT were 138±7.62 bpm, 59±3.24 mm, and 2.3±0.61 mm respectively. PAPP-A and AFP significantly (p<0.05) correlated with fetal HR, CRL, and NT at 11±1 weeks of gestation. The mean ratio of AFP:PAPP-A in low-risk pregnancies was 3.37.

Conclusions: The maternal serum biochemical attributes correlated well with the fetal ultrasound scans. The findings of the present study can prove to be clinically useful for clinical research, obstetrics, and gynaecology, especially for examinations of first-trimester pregnancies.

## Introduction

The first trimester of pregnancy is the recommended time for screening for major anomalies and abnormalities of the chromosomes [1]. In women with atypical karyotype, structural malformations and fetal death are linked to nuchal translucency (NT) between 10 and 12 weeks of gestation [2]. The pregnancy-associated plasma protein-A (PAPP-A) functions like insulin-like growth factor and, therefore, it is critical to explore the function of PAPP-A in early fetal development [3]. The first-trimester screening can be used for the noninvasive detection of aneuploidy and, recently, significant research has suggested that PAPP-A could predict future perinatal complications, specifically growth abnormalities in fetuses [4]. The proteolytic function in PAPP-A is activated upon collagen binding, and it plays a pivotal role in bone remodeling and wound healing [5]. The alpha fetoprotein (AFP) gene on chromosome 4q25 encodes a protein that is produced by the yolk sac of the embryo and the liver of the developing fetus. AFP levels in serum, amniotic fluid, and urine can be used as a screening test for congenital disabilities, chromosomal disorders, and some types of tumors and pathology in adults [6]. As the prenatal levels of AFP begin to rise at the end of the first trimester and decrease after several weeks of gestation, maternal serum AFP forms part of screening tests for fetal anomalies [7]. Numerous previous studies have shown that higher NT is also linked to a poorer outcome for the fetus. Unfortunately, long-term prognoses for these infants are not clear. The aim of this study was to evaluate the correlation between the maternal blood biochemical parameters PAPP-A and AFP with ultrasound scans during early pregnancy in the first trimester.

## Materials and methods

Participant recruitment

The observational prospective study included 139 women with live fetuses between 11±1 weeks of gestation and who had no significant vaginal bleeding (spotting or a few drops of blood) referred from the Department of Obstetrics and Gynaecology, Indira Gandhi Institute of Medical Sciences (IGIMS), Patna, Bihar, India. They were selected from healthy full-term singletons and remained healthy from the time of onset of pregnancy to the time of testing.

The study was approved by the IGIMS institutional ethics committee (no. 682/IEC/IGIMS/2018; dt: 21/12/2018). Written informed consent was obtained from the participants before participation in the study.

Inclusion and exclusion criteria

Women aged between 21 to 35 years, in their first trimester of pregnancy, with no history of abnormal pregnancy, and with a normal screening for fetal NT using color duplex ultrasound between 11±1 weeks were included in the study. However, pregnant women with history of tumor, degenerative disease, myopathy, organ transplant, alcohol consumption, smoking or any substance abuse such as chewing tobacco, receiving recent blood transfusion, women with endocrinopathies, chronic infections, or other pathological conditions were excluded from the study.

Assessment of risk of fetal chromosomal abnormalities

The risk of fetal chromosomal abnormalities at first trimester of pregnancy was analyzed by VeriSeq NIPT Solution v2 platform (Illumina, San Diego, CA, USA) for noninvasive prenatal testing (NIPT) assay. This technique relies on the analysis of the circulate cell-free fetal DNA (cfDNA) to know whether any risk of fetal chromosomal abnormalities during first-trimester pregnancy [8].

The cfDNA was extracted from the maternal peripheral whole blood by cfDNA extraction kit (MagMAX Cell-Free DNA Isolation Kit; Applied Biosystems, Waltham, MA, USA). This was followed by sample preparation and next generation whole genome sequencing by Illumina platform. The paired-end sequencing data was analysed by the Illumina VeriSeq NIPT Assay Software. This test can detect aneuploidies of autosomes 21, 18, 13, sex chromosomes (X0, XXX, XXY, and XYY), and rare autosomal aneuploidies [9]. The Assay Software assessed the log likelihood ratio (LLR) for each target chromosome and each sample to provide a determinant of aneuploidy. The LLR is the probability of a sample being affected by the observed coverage versus the probability of a sample being unaffected given the same observed coverage [10]. Table 1 represents the high-risk of trisomy and high-risk LLR cut-offs of monosomy line for aneuploidies of clinical indication for NIPS by the Illumina VeriSeqTM NIPT assay software. Table 2 represents the sensitivity and specificity of the trisomies and aneuploidies. Further validation was performed at Med Genome Labs Ltd. (Bangalore, India).

**Table 1:** High-risk of trisomy and high-risk LLR cut-offs of monosomy line for aneuploidies of clinical indication for NIPS by the Illumina VeriSeqTM NIPT assay software LLR: log likelihood ratio, NIPT: noninvasive prenatal testing

Chromosome No.	High-Risk LLR cut-off (Trisomy)	High-Risk LLR cut-off (Monosomy)
Chromosome 1	≥ 7	≥13.2
Chromosome 2	≥9	≥13.6
Chromosome 3	≥5	≥13.8
Chromosome 4	≥7	≥15.2
Chromosome 5	≥7.6	≥17
Chromosome 6	≥7.3	≥15.4
Chromosome 7	≥6.6	≥14
Chromosome 8	≥5.8	≥14.8
Chromosome 9	≥8	≥13.6
Chromosome 10	≥8.8	≥14.7
Chromosome 11	≥12.2	≥15.7
Chromosome 12	≥11.6	≥12.8
Chromosome 13	≥3	≥16.5
Chromosome 14	≥12.7	≥14.7
Chromosome 15	≥9.8	≥16.4
Chromosome 16	≥10.7	≥15.3
Chromosome 17	≥16.8	≥15.7
Chromosome 18	≥3	≥11.3
Chromosome 19	≥15.5	≥27.5
Chromosome 20	≥10.6	≥18.2
Chromosome 21	≥2.5	≥13.2
Chromosome 22	≥13.5	≥15.3

**Table 2:** Sensitivity and specificity for trisomies 21, 18, 13, sex chromosomal aneuploidies, and rare autosomal aneuploidies * Confidence Interval (CI) is based on Wilson's score method.

Sensitivity and specificity	Trisomy 21	Trisomy 18	Trisomy 13	RAA	Any anomaly
Sensitivity	>99.9%	>99.9%	>99.9%	96.4%	95.5%
Specificity	99.9%	99.90%	99.90%	99.8%	99.34%

Ultrasonography

Ultrasonography was performed by experienced radiologists using ultrasound machine (USS-H60; Samsung, Suwon, Korea, 100-240V, 620VA, 50/60Hz) available in the institute. A transabdominal and transvaginal ultrasound examination with variable focus and curvelinear transducer and a transvaginal transducer was performed in all cases. At 11±1 weeks of gestation, the fetal NT thickness, fetal heart rate (FHR), and crown rump length (CRL) were measured by experienced ultrasound examiners according to the standards of the Fetal Medicine Foundation [11,12].

Biochemical analysis

Phlebotomy was performed by venipuncture and blood samples were collected in plain vials in order to examine AFP and PAPP-A levels. Samples were processed within four hours of collection in a refrigerated centrifuge and stored at −80°C until analysis. PAPP-A and AFP levels were retrieved from the patients’ clinical records. The PAPP-A and AFP levels were measured by fully automated chemiluminescent immunoassay (Thyrocare Technologies Limited, Mumbai, India).

Statistical analysis

Statistical analysis was performed using the SPSS software package version 22 (IBM Corp., Armonk, NY, USA). Data was represented as mean±SD. The PAPP-A and AFP were log-transformed and the maternal blood markers were tested parametrically because they resembled Gaussian distributions. Pearson's correlation coefficients were calculated to evaluate the correlation between PAPP-A, AFP, FHR, CRL, and NT. P values less than 0.05 were considered to be significant.

## Results

The mean age of the participants was 28.35±3.87 years (minimum=21, maximum=35). None of the women were at risk of fetal chromosomal abnormalities. The mean AFP level in the maternal plasma was 14.76 ±1.04 ng/mL and the mean PAPP-A level was 4.37±0.86 mIU/ml. The mean basal metabolic rate (BMR) was 5570.55±86.79 kJ/24 h and the mean BMI was 21.60±1.43 kg/m2 (Table 3).

**Table 3:** First-trimester biochemical marker levels in the first trimester pregnant women AFP: alpha fetoprotein, PAPP-A: pregnancy-associated plasma protein-A, BMR: basal metabolic rate, BMI: body mass index. Values are presented as mean±SD.

Parameters	mean±SD
AFP (ng/mL)	14.76±1.04
PAPP-A (mIU/mL)	4.37±0.86
BMR (kJ/24 h)	5570.55±86.79
BMI (kg/m^2^)	21.60±1.43

The ratio of the mean AFP:PAPP-A was observed to be 3.37 in the first trimester for low-risk NIPT. The mean FHR was found to be 138±7.62 bpm, the mean CRL was 59±3.24 mm, and the mean NT was to be 2.3±0.61 mm (Table 4).

**Table 4:** The FHR, CRL, and NT in the first trimester by sonographic measurements. FHR: fetal heart rate, CRL: crown rump length, NT: nuchal translucency. Values are presented as mean±SD.

Fetal ultrasonographic parameters	mean±SD
FHR (bpm)	138±7.62
CRL (mm)	59±3.24
NT (mm)	2.3±0.61

Maternal AFP levels showed a significant positive correlation with FHR (r=0.35, p=0.02), CRL (r=0.40, p=0.01), and NT (r=0.36, p=0.01). Similarly, the maternal PAPP-A levels showed a significant positive correlation with FHR (r=0.45, p=0.01), CRL (r=0.41, p=0.02), and NT (r=0.33, p=0.01) (Table 5).

**Table 5:** Pearson’s correlation analysis between the levels of maternal pregnancy-associated plasma protein-A (PAPP-A), alfa-fetoprotein (AFP), and ultrasound indices (FHR, CRL, and NT). AFP: alpha fetoprotein, PAPP-A: pregnancy-associated plasma protein-A, FHR: fetal heart rate, CRL: crown rump length, NT: nuchal translucency. Values are presented as Pearson’s correlation coefficient (r) and p value. P values less than 0.05 were considered to be statistically significant.

Attribute	FHR (bpm)	CRL (mm)	NT (mm)
AFP (ng/mL)	0.35 (0.02)	0.40 (0.01)	0.36 (0.01)
PAPP-A (mIU/mL)	0.45 (0.01)	0.41 (0.02)	0.33 (0.01)

## Discussion

The present study evaluated the correlation of maternal PAPP-A and AFP levels, which are the most widely used markers of fetal growth in the first trimester of pregnancy, with fetal sonographic attributes such as HR beats, CRL, and NT. It has been reported that a low plasma level of PAPP-A may be a biochemical marker for pregnancies with contorted fetuses, and low PAPP-A may be associated with prenatal screening for chromosomal syndrome [13]. Low levels of PAPP-A may also indicate adverse complications in the placenta, such as intrauterine growth restriction, preeclampsia, placental abruption, and premature birth [14]. However, AFP levels are linked to useful diagnostics in the detection of fetal abnormalities, particularly neural-tube defects [15]. 

Our results provide data that is useful for making suggestions for pregnant patients undergoing first-trimester ultrasounds. We demonstrated that measurements of fetal sonographic findings at 11±1weeks of gestation are significantly associated with maternal serum levels of PAPP-A and AFP. In the first trimester, levels of PAPP-A were associated with pregnancy outcomes indicative of placental discombobulation. Our work has shown that the ratio of the mean of AFP:PAPP-A is 3.37 in the first trimester for low-risk NIPT, which is in line with the findings of several other studies [16,17]. Another study also reported that a ratio of AFP:PAPP-A < 10 is associated with no-risk fetal status [18]. Therefore, the AFP:PAPP-A ratio of 3.37 observed in the present study is indicative of no-risk fetal status in the first trimester of pregnancy.

The fetal HR beats, CRL, and NT parameters correlated well with the maternal plasma PAPP-A and AFP levels, consistent with the findings of several other studies [19,20]. In the human pregnancies studied to date, it has been observed that abnormalities in placental development may affect the amount of PAPP-A released into the maternal circulation, therefore, PAPP-A levels could be considered the marker of fetal growth [21,22]. However, there is a scarcity of literature on the dynamics of PAPP-A between the fetus and the mother. Although PAPP-A has been reported in embryonic and fetal tissues, in amniotic and coelomic fluids, only one study has reported its levels in the fetal blood [23]. It is, therefore, possible that PAPP-A made in the placenta is partly transferred to the fetal circulation, as well as that PAPP-A made in the maternal circulation may have some relationship with the fetal growth. We, therefore, suggest that a positive association observed between the PAPP-A and ultrasonographic scan findings in the first trimester of pregnancy is indicative of the existence of a normal fetal growth. Furthermore, there is the possibility that the correlation between maternal PAPP-A and CRL is just a reflection of fetal growth, as previous studies have identified the correlation between maternal PAPP-A and fetal maximum length in women at similar gestational ages [22,24]. Hence, CRL may simply be an outcome of overall growth. PAPP-A and CRL measurements are some of the most widely used growth indicators at this gestational age and were analyzed in this study, thus the correlation observed is specific and independent of overall fetal development.

## Conclusions

The maternal PAPP-A and AFP correlated well with fetal ultrasound scans in the first trimester of pregnancy. Since the PAPP-A and AFP levels at 11±1 weeks of gestation are significantly related to the FHR, CRL, and NT, they might regulate the physiological and physical attributes for low-risk fetuses.
